# Inflammatory biomarker changes in healthy adults secondary to electronic cigarette use: A scoping review

**DOI:** 10.1002/iid3.1170

**Published:** 2024-02-01

**Authors:** Shawn Boss, Michael Bertolio, Laura Lipke

**Affiliations:** ^1^ Kirksville College of Osteopathic Medicine A.T. Still University Kirksville Missouri USA; ^2^ Science Library Binghamton University Binghamton New York USA

**Keywords:** biomarkers, cardiovascular disease, e‐cig, electronic cigarettes, inflammation, oxidative stress, pulmonary disease, vape

## Abstract

**Context:**

There has been a global increase in the use of electronic cigarettes (EC). However, to our knowledge, no review has summarized or categorized changes in inflammatory biomarkers after EC use in the extant literature.

**Objective:**

To evaluate changes in general, cardiopulmonary, and oxidative stress‐related inflammatory biomarkers in healthy adults who use ECs.

**Methods:**

A scoping review was conducted according to the Arksey and O'Malley framework. PubMed and MEDLINE (Ovid) databases were used for our search. After initial pilot searches and discussions, we performed a final search with medical subject headings and plain language terms related to inflammation, biomarkers, ECs, and adult humans. All full‐text articles, gray literature, and primary studies dating from the inception of the searched databases to the present were included. Studies of human participants with known confounding medical histories were excluded.

**Results:**

Thirty‐seven studies met the inclusion criteria. After short‐term (<1 month) use, ECs containing nicotine moderately increased cardiovascular (CV) and oxidative stress markers of inflammation. Of all reported results, 50% of CV biomarkers were increased, and 64% of oxidative stress markers were increased. After long‐term (>1 month) use, ECs containing nicotine produced mixed results. Two commonly measured biomarkers in this group, matrix metalloproteinase‐9 (MMP‐9) and interleukin‐6 (IL‐6), were elevated in 75% and 60% of measured instances, respectively.

**Conclusion:**

The results of studies evaluated in our scoping review suggested that short‐term use of nicotine‐containing ECs may result in increased CV and oxidative stress inflammation, contributing to potential CV or neurologic disease development. The results of studies evaluated in our scoping review also suggested that long‐term use of nicotine‐containing ECs resulted in no significant changes in general inflammatory biomarker levels. A rigorous systematic review and meta‐analysis is necessary to corroborate our findings and to determine the effect of long‐term EC use on MMP‐9 and IL‐6 levels.

## Graphical abstract

The current review summarizes changes in inflammatory biomarkers in adults after the use of electronic cigarettes. Cardiovascular, pulmonary, general, and oxidative‐stress biomarkers reported in the extant literature on this topic are reviewed.

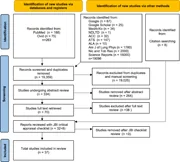

## 1 INTRODUCTION

Electronic cigarettes (EC), also known as e‐cigarettes or vapes, have been popular among US adults and adolescents since 2006.^1^, ^2^ These devices are designed to heat nicotine, flavoring additives, and vegetable glycerol or propylene glycol for users to inhale in an aerosolized state. As such, ECs come in several flavors and mostly imitate the taste of various fruits, drinks, or candies. They are marketed by EC companies as a safer alternative to traditional tobacco cigarettes.^3^, ^4^ However, their long‐term effects on user health are unknown. Case reports and studies have reported lung injury, cardiovascular (CV) disease, and strokes are associated with EC use.^5^, ^6^, ^7^ The use of EC use has also been associated with myocardial infarctions, which suggests they are not as safe as companies would like consumers to believe.^8^, ^9^ Additionally, the electronic nicotine delivery systems of ECs have been shown to contain harmful metals, such as arsenic, chromium, nickel, and lead.^9^ Despite these reports, the EC industry has continued to grow. In the United States alone, the EC vaping industry reached a market value of $20.4 billion in 2021 and is expected to reach a value of $30 billion by 2027.^10^ Therefore, healthcare professionals and researchers have become more concerned about the potential consequences of EC use.

Traditional cigarettes have been repeatedly correlated with poor health outcomes^11^ and particularly with increased inflammation and development of CV disease, strokes, aortic aneurysms, peripheral vascular disease, and various cancers.^12^ With the widespread use of EC, it is important to determine whether users are at increased risk of developing similar conditions. To our knowledge, no review has summarized, categorized, and reported changes in inflammatory biomarkers after EC use in the extant literature. Such information would be vital for future investigations of the exact risks posed by ECs. Therefore, the purpose of the current scoping review was to summarize the findings of selected studies and report changes in general, cardiopulmonary, and oxidative stress‐related inflammatory biomarkers in healthy adults who use ECs.

## 2 METHODS

The current scoping review was conducted according to the Arksey and O'Malley framework for scoping reviews and reported according to the 2018 PRISMA extension for scoping reviews.^13^, ^14^ Our inclusion criteria were designed to include the maximal amount of primary studies to summarize changes in inflammatory biomarkers in healthy adults who use ECs while accounting for their health history and age‐related illnesses. Only studies of human participants with no known confounding medical history were included in the review; animal studies were excluded to eliminate potential confounding variables. Further, only studies that investigated adult participants and measured biomarkers through in‐vivo serum collections, imaging, urinalysis, or breath test were included. Primary studies from the inception of PubMed and MEDLINE (Ovid) to the present were included to ensure all available data would be considered. Likewise, we searched the gray literature (Google Scholar, MedArXiv, NDLTD, and OATD), including conference posters and abstracts, and clinical trials to maximize the number of reviewed studies. Investigators also hand‐searched three relevant journals. Our search was limited to English language studies because of translation limitations.

Before conducting any literature searches, we performed a preliminary search of PubMed, MEDLINE (Ovid), the Cochrane Database of Systematic Reviews, and the *JBI Evidence Synthesis* journal in August 2022 to identify any current systematic or scoping reviews on this topic. None were identified. Next, we performed a pilot search of PubMed and MEDLINE (Ovid) and identified 460 potential articles. Therefore, we determined a scoping review would be appropriate for this topic because it provides the best framework for reporting on relevant research and may serve as a reference for future systematic reviews and meta‐analyses.

Our final search strategy was conducted in July 2023 and based on exemplar articles identified in PubMed and MEDLINE (Ovid), the associated plain language, medical subject headings terms, and terms predetermined by study investigators were used to develop the initial search strategy in PubMed and then translated to MEDLINE (Ovid). (Appendices A and B). A simplified version of the database search was used to identify gray literature in the previously stated sources.

**Figure 1:**
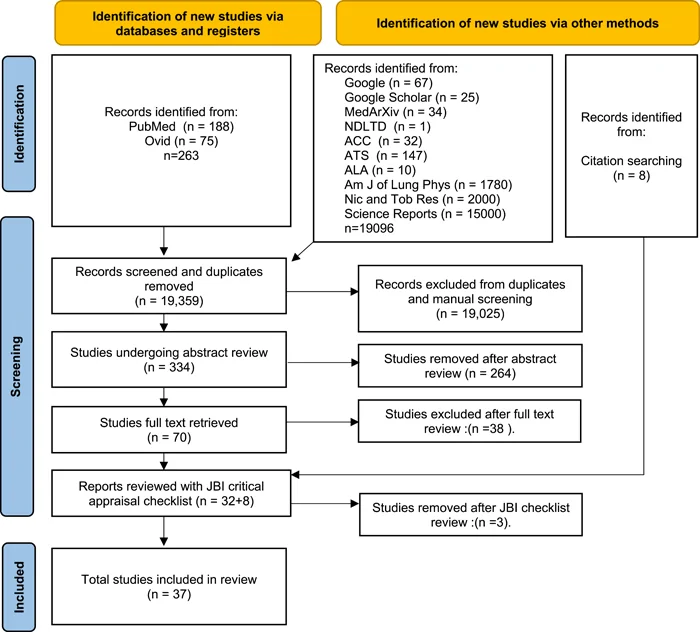
PRISMA flow diagram. American College of Cardiology (ACC), American Lung Association (ALA), American Thoracic Society (ATS), Networked Digital Library of Theses and Dissertations (NDLTD).

After these initial searches, we identified three journals that published the most relevant studies: *American Journal of Physiology—Lung Cellular and Molecular Physiology*, *Nicotine and Tobacco Research*, and *Science Reports*. Study investigators independently screened article titles and abstracts from these journals. All journal issues from 2014 to 2022 were hand‐searched. Initially, investigators did not compare search findings to ensure independent assessment. Additionally, three relevant professional organizations (American College of Cardiology, American Thoracic Society, and American Lung Association) were identified, and their websites were screened separately by study investigators for any relevant literature. Finally, a last literature search was conducted in Google, Google Scholar, the Networked Digital Library of Theses and Dissertations, and medRxiv for any articles or gray literature that was not identified in previous searches. A comprehensive history of article screening is presented in Figure 1.

All searches were imported into Zotero citation management software and then exported to Covidence review screening software and duplicates were removed.^15^, ^16^ Study investigators (S. B. and M. B.) independently screened articles by titles and abstracts. Disagreements were settled through discussion. A full‐text review of articles was then performed in a similar fashion. Again, any disagreements were resolved through discussion. After the list of relevant studies was finalized, S. B. performed “citation chasing” on 32 articles. A full list of references from each article was screened independently by title and abstract using Covidence.^16^ Eight additional articles were identified as potentially relevant and underwent full‐text review as described above.

To ensure quality, all articles that underwent full‐text review were also appraised independently by S. B. and M. B. for validity and bias using the JBI critical appraisal checklist. Decisions to remove a study based on the JBI critical appraisal criteria were discussed between investigators and mutually decided through discussion.^17^ After appraising the quality of the 40 identified articles for review, three were removed. One article was removed for an inappropriate study design, unclear outcome assessment, and generally unclear methodology. Another article was excluded for unreliable measurement methods and unclear description of study participants and settings. The third article was removed because it used a much smaller control group than the experimental group. Following this, a final list of studies for review was compiled.

Data were extracted from included studies using a template created by study investigators. The template was largely based on JBI recommendations with some alterations made by S. B. and M. B. (Table C1). In addition to the template, we also used the data extraction tool in Covidence. Independent extraction was performed by S. B. and M. B., and data were later compared to ensure consistency during extraction.

**Figure 2:**
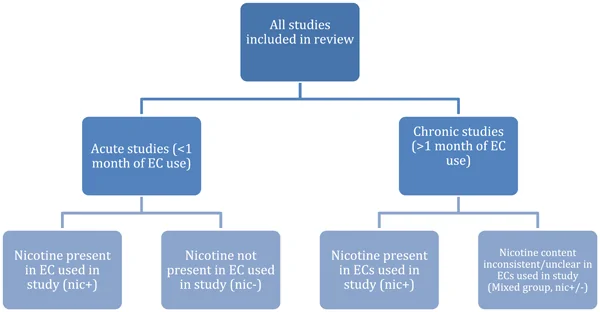
Classification system for reviewed studies. Reviewed studies were sorted into the above categories to better assess changes in inflammatory biomarkers. Studies were assigned to either acute (<1 month of use) or chronic (>1 month of use) based on the duration of electronic cigarettes use by study participants.

In an effort to succinctly categorize the results of reviewed studies, the 37 articles were assigned to the following EC use classification: short‐term (acute) studies using nicotine‐containing (nic+) devices, acute studies using nicotine‐free (nic−) devices, long‐term (chronic) studies using nic+ devices, and chronic studies using nic− devices. Studies could be assigned to only one category or more than one category. An example of this would be a study that required participants to use both a nic+ EC and a nic− EC for an hour in separate instances. In this example, the study would be assigned to both the short‐term nic+ and short‐term nic− categories. For the current review, acute studies were defined as participant use of ECs for <1 month. Chronic studies were defined as participant use of ECs for more than 1 month. Reviewed studies were assessed to determine whether participants used ECs that were nic+, nic−, or both. Some studies collected biomarker measurements after participants nic+ or nic− use on separate occasions. In these instances, results were documented in both categories. For example, if a study measured serum nitric oxide in participants after nic+ and nic− use, two results were documented (one for acute nic+ and one for acute nic− categories). Chronic studies that did not clearly indicate the nicotine exposure of participants were grouped into a chronic nic+/− category; this unspecified category was used to mitigate potential confounding variables between participants and study methods (Figure 2).

The biomarkers assessed in the current scoping review were categorized into four broad biomarker categories: CV, pulmonary, general inflammation, and oxidative stress. All biomarkers assessed in the 37 reviewed studies were checked against the current extant literature for documented evidence that they were implicated in proinflammatory processes. Biomarkers were excluded if they were not implicated in proinflammatory processes. If the extant literature stated that a biomarker had both inflammatory and anti‐inflammatory characteristics, it was excluded. S. B. and M. B. agreed upon the comprehensive list of inflammatory biomarkers to be included in the current review and excluded any biomarkers from the current review's results that did not meet the inclusion criteria. The complete list of inflammatory biomarkers assessed in the current scoping review by category are presented in Table 1.

**Table 1:** Assessed biomarkers included in the current scoping review for acute and chronic EC use.

General		Cardiovascular	Pulmonary	Oxidative stress
ASC	IL‐2	CD144 (endothelial‐derived microvesicle)	18F‐NOS BPND	13‐HODE
Azurocidin 1	IL‐5	E‐selectin	CC16	8‐isoprostagland‐PGFalpha/8‐isoprostane
Calprotectin	IL‐6	EC derived EVs (PS + CD62E + )	FeNO	9‐HODE
CARD	IL‐7	EMP CD42b − CD31+	MMP‐2	ApoB oxidized
Caspase‐1	IL‐8	EMPs	MMP‐9	MMP‐9
CD14 (microvesicle‐derived by monocytes)	LTB4	ICAM‐1	MUC5AC	COS B cells
CD45 (microvesicle‐derived by leukocytes)	MCP‐1	Nitric oxide metabolites	MUC5B	COS neutrophil cells
Coronin‐1	Microsemionoprotein beta	P‐selectin/sP‐selectin	Neutrophil elastase	COSin NK cells
CRP	MIP1alpha	PECAM	YKL‐40	COS in T cells
CXCL1	MIP1B	Platelet‐derived EVs (PS + CD41 + )	CC10	COS in monocytes
CXCL2	Nucleobindin‐1	Platelet‐derived EVs expressing P‐selectin		COS in CD45 cells
EN‐rage	PAI‐1	PMP (CD31 + CD42 + )		H2O2
Eotaxin	PAD4	sCD‐40L/CD‐40L		Homocitrulline/lysine ratio
Fibrinogen	Proteinase 3	Serum NO		Low‐density lipoprotein oxidizability
Fit1	RAGE	VCAM‐1		MDA
Galectin‐3	RANTES	EV (PS + CD41 + + CD62P)		MPO
GM‐CSF	S100A8	Thromboxane B2		Protein‐bound 3‐chlorotyrosine/tyrosine ratio
HMGB‐1	S100A9			sNOX2‐dp
Interferon‐γ	SYTO 13 dye			4‐hydroxynonenal
IL‐12p70	TNF‐alpha			8‐oxo‐dG
IL‐17a	TNF‐beta			sPLA2
IL‐1alpha	Uric acid			
IL‐1beta				

Abbreviations: 18F‐NOS BPND, 18F‐6‐(1/2)(2‐fluoro‐propyl)‐4‐methylpyridin‐2‐amine nondisplaceable binding potential; ASC, apoptosis‐associated speck‐like protein; CARD, caspase activation recruitment domain; CC16, Clara cell secretory protein; COS, cytoplasmic oxidative stress; CRP, C‐reactive protein; EC, e‐cigarette; EMPs, endothelial microparticles; EN, extracellular newly identified; EV PS, platelet‐derived extracellular vesicles; FeNO, fraction of exhaled nitric oxide; GM‐CSF, granulocyte monocyte colony stimulating factor; HMGB‐1, high‐mobility group box 1; HODE, hydroxyoctadecadienoic acid; ICAM, intracellular adhesion molecule; IL, interleukin; LTB4, leukotriene B4; MCP‐1, monocyte chemoattractant protein‐1; MDA, malondialdehyde; MMP, matrix metalloproteinase; MPO, myeloperoxidase; MUC5AC, mucin 5AC; NK, natural killer; NO, nitric oxide; PAD4, protein arginine delaminate 4; PAI‐1, plasminogen activator inhibitor 1; PECAM, platelet endothelial cell adhesion molecule; PMP, platelet microparticle; RAGE, receptor for advanced glycation endproducts; RANTES, regulated upon activation, normal T cell expressed and presumably secreted; sPLA2, secretory phospholipase A2; TNF, tumor necrosis factor; VCAM, vascular cell adhesion molecule.

Once the final list of inflammatory biomarkers was determined, statistically significant increases or decreases between the experimental and control group or baseline measurements were recorded for the reviewed articles. Any reported results of no statistically significant difference were recorded as “no change.” Biomarkers measured after nic+ and nic− use were sorted into the appropriate categories for our review and could be counted in more than one category. Many biomarkers were found and measured multiple times, and some were only reported in a single study. For example, if a study measured three different biomarkers after nic+ and nic− use, then those three biomarkers were included in both the nic+ and nic− results of our review. All biomarkers were analyzed using simple frequency and percentage within their respective categories.

Biomarkers analysis was performed using simple frequency and percentage. The primary purpose of the current review was to summarize and report changes in inflammatory biomarkers after EC use; therefore, no additional methods of analysis were performed. After the result of a study was organized into the short‐term or long‐term use categories, it was sorted into nic+ or nic−, and finally into general inflammation, CV, pulmonary, or oxidative stress. The frequency of documented increases, decreases, or no changes in biomarker levels were then divided by the total number of results in that category.

## 3 RESULTS

### 3.1 Acute studies with nicotine‐containing EC devices

**Figure 3:**
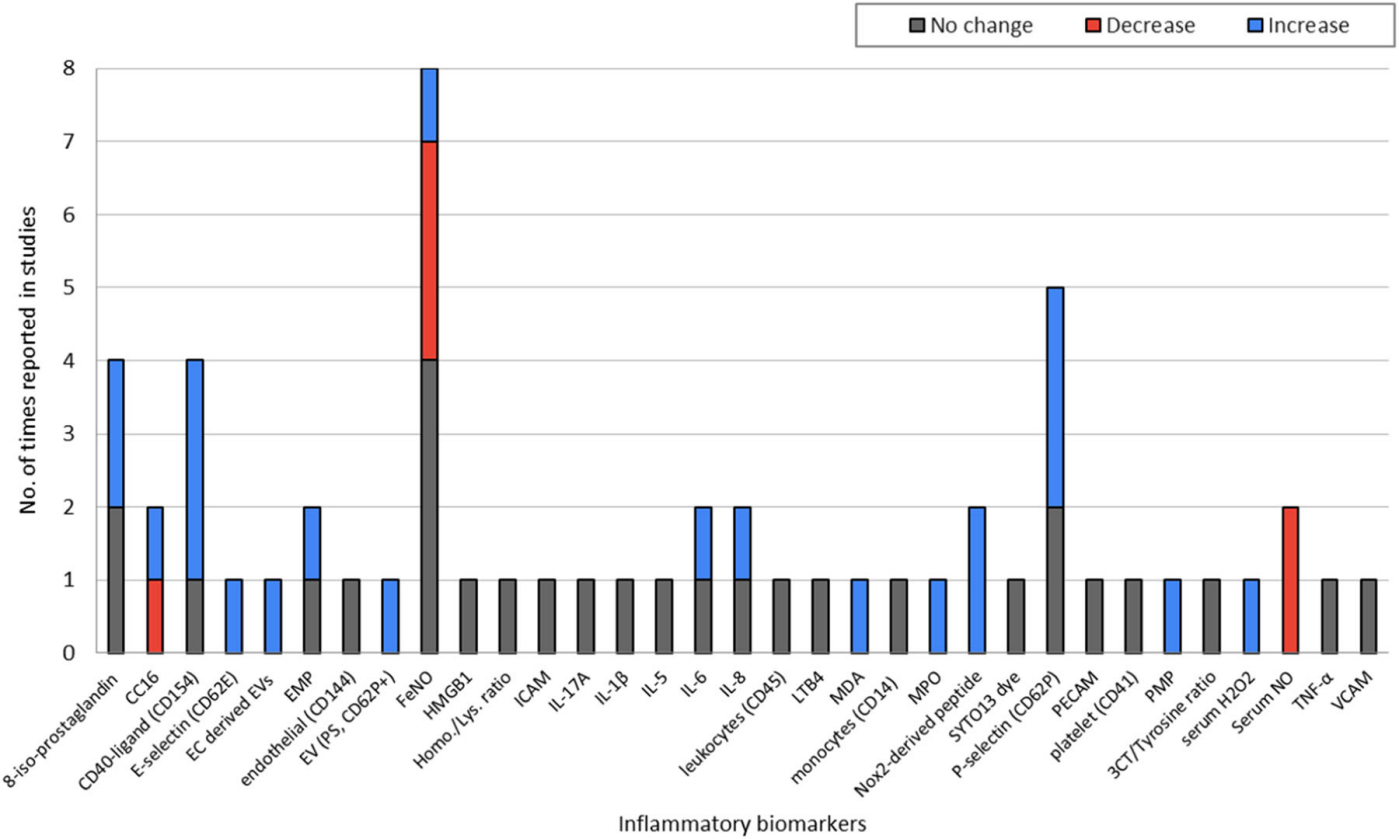
Inflammatory biomarker changes for reviewed acute studies with the use of nicotine‐containing e‐cigarette (EC) devices. Participants using ECs for <1 month were compared with baseline measurements or control groups. Each reported statistically significant increase or decrease was counted separately for a biomarker, and no statistically significant differences were counted as no change. CC16, Clara cell protein 16; EC, endothelial cell; EMP; endothelial microparticles; EV, extracellular vesicles; FeNO, fraction of exhaled nitric oxide; HMGB‐1, high mobility group box 1 protein; ICAM, intracellular adhesion molecule; IL, interleukin; LTB, leukotriene; MDA, malondialdehyde; MPO, myeloperoxidase; PECAM, platelet endothelial cell adhesion molecule; PMP, platelet microparticle; TNF‐α, tumor necrosis factor alpha; VCAM, vascular cell adhesion molecule.

Of the 37 reviewed studies, 19 studies measured inflammatory biomarkers in the acute nic+ category (Figure 3). Fraction of exhaled nitric oxide (FeNO) was the most commonly measured marker of inflammation in this category of studies. FeNO was reported a total of eight times in acute nic+ studies, with four (50%) reporting no change, three (38%) a decrease, and one (13%) an increase in inflammation. In reviewed studies, the use of an EC increased 22 (39%) of 56 biomarkers and decreased six (11%); there was no change in 28 (50%). Of 22 results from studies measuring CV biomarkers, 11 (50%) increased, two (9%) decreased, and nine (41%) had no change. Of 10 results on pulmonary biomarkers, two (20%) increased, four (40%) decreased, and four (40%) had no change. Of the 13 results on general inflammatory biomarkers, two (15%) increased, and 11 (85%) had no change. Of the 11 oxidative stress biomarker reports, seven (64%) increased, and four (36%) had no change. A complete list of reviewed studies and measured biomarkers for this EC use category is presented in Table D1.

### 3.2 Acute studies without nicotine‐containing EC devices

**Figure 4:**
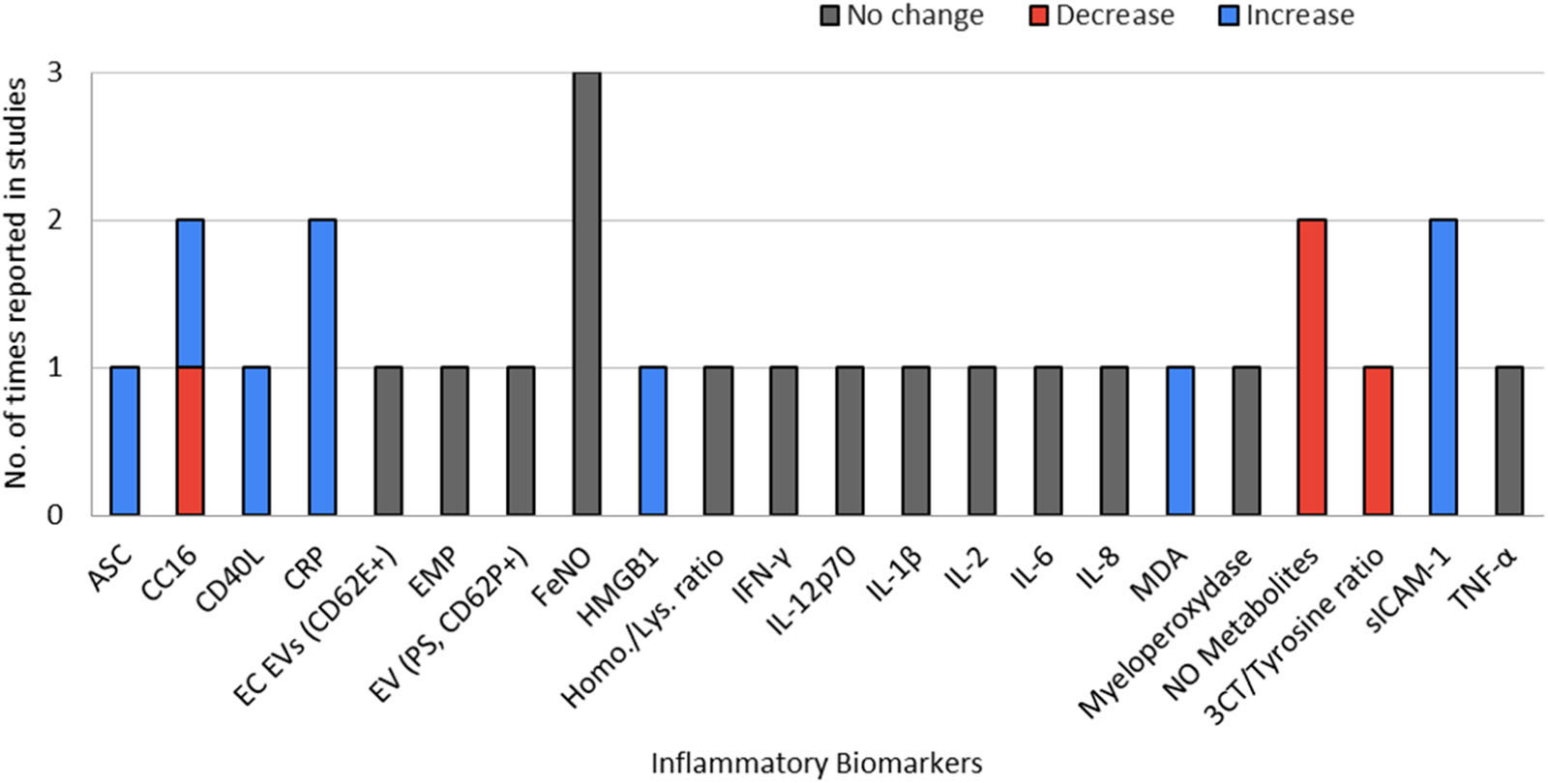
Inflammatory biomarker changes for reviewed acute studies without the use of nicotine‐containing e‐cigarette (EC) devices. Participants using ECs for <1 month were compared with baseline measurements or control groups. Each reported statistically significant increase or decrease was counted separately for a biomarker, and no statistically significant differences were counted as no change. 3CT/Tyrosine, protein‐bound 3‐chlorotyrosine/tyrosine ratio; ASC, apoptosis‐associated speck‐like protein; CC16, Clara cell secretory protein; CD40L, CD40 ligand; CRP, C‐reactive protein; EC EVs, endothelial cell‐derived extracellular vesicles; EMP, endothelial microparticles; EV PS, platelet‐derived extracellular vesicles; FeNO, fraction of exhaled nitric oxide; HMGB‐1, high mobility group box 1 protein; Homo./Lys., homocysteine to lysine ratio; IFN‐γ, interferon gamma; IL‐12p70, interleukin 12p70; IL‐1B, interleukin 1 beta; IL‐2, interleukin 2; IL‐6, interleukin 6; IL‐8, interleukin 8; MDA, malondialdehyde; MPO, myeloperoxidase; NO, nitric oxide; sICAM‐1, soluble intercellular adhesion molecule 1; TNF‐α, tumor necrosis factor alpha.

Twelve of the 37 reviewed studies measured inflammatory biomarkers in the acute nic− category (Figure 4). FeNO was the most measured biomarker in this category of studies as well. FeNO was reported a total of three times across studies in this category. All three (100%) results reported no change in inflammation. In these reviewed studies, use of EC increased nine (32%) of 28 biomarkers and decreased four (14%); there was no change in 15 (54%). Of the eight results on CV biomarkers, three (38%) increased, two (25%) decreased, and three (38%) had no change. Of the five results on pulmonary biomarkers, one (20%) increased, one (20%) decreased, and three (60%) had no change. Of the 11 results on general inflammatory biomarkers, four (36%) increased, and seven (64%) had no change. Of the five results on oxidative stress biomarkers, two (40%) increased, one (20%) decreased, and two (40%) had no change. A complete list of reviewed studies and measured biomarkers for this EC use category is presented in Table D2.

### 3.3 Chronic studies with nicotine‐containing EC devices

**Figure 5:**
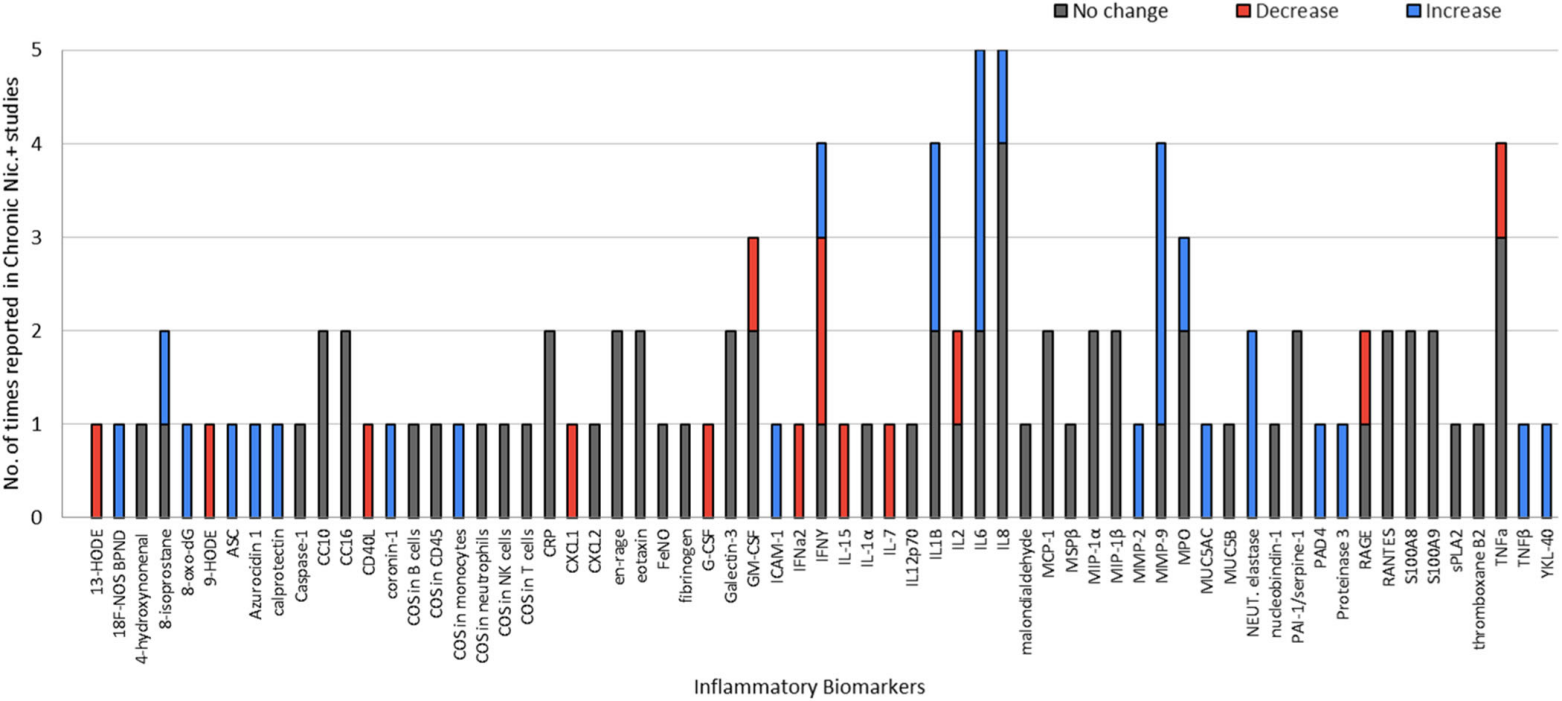
Inflammatory biomarker changes for reviewed chronic studies with the use of nicotine‐containing e‐cigarette (EC) devices. Participants using ECs for more than 1 month were compared with baseline measurements or control groups. Each reported statistically significant increase or decrease was counted separately for a biomarker, and no statistically significant differences were counted as no change. 8‐oxo‐dG, 8‐Oxo‐2′‐deoxyguanosine; 9‐HODE, 9‐Hydroxyoctadecadienoic acid; 13‐HODE, 13‐Hydroxyoctadecadienoic acid; 18F‐NOS BPND, 18F‐6‐(1/2)(2‐fluoro‐propyl)‐4‐methylpyridin‐2‐amine nondisplaceable binding potential; ASC, apoptosis‐associated speck‐like protein; CC10, Clara cell protein 10; CC16, Clara cell secretory protein; CD40L, CD40 ligand; COS, cytoplasmic oxidative stress; CRP, C‐reactive protein; CXCL1, chemokine (C‐X‐C motif) ligand 1; CXCL2, chemokine (C‐X‐C motif) ligand 2; FeNO, fraction of exhaled nitric oxide; G‐CSF, granulocyte colony stimulating factor; GM‐CSF, granulocyte monocyte colony stimulating factor; ICAM‐1, intracellular adhesion molecular 1, IFNα2, interferon alpha 2; IFN‐γ, interferon gamma; IL‐1a, interleukin 1 alpha; IL‐15, interleukin 15; IL‐17, interleukin 17; IL‐12p70, interleukin 12p70; IL‐1B, interleukin 1 beta; IL‐2, interleukin 2; IL‐6, interleukin 6, IL‐8, interleukin 8; MCP‐1, monocyte chemoattractant protein‐1; MIP‐1a, macrophage inflammatory protein‐1 alpha; MIP‐1beta, macrophage inflammatory protein‐1 beta; MMP‐2, matrix metalloproteinase‐2; MMP‐9, matrix metalloproteinase‐9; MPO, myeloperoxidase; MSPB, microseminoprotein beta; MUC5AC, mucin 5AC; MUC5B, mucin 5B; NEUT elastase, neutrophil elastase; PAI‐1, plasminogen activator inhibitor 1; PAD4, peptidyl arginine deiminase 4; RAGE, receptor for advanced glycation endproducts; RANTES, regulated upon activation, normal T cell expressed and presumably secreted; S100A8, S100 calcium‐binding protein A8; S100A9, S100 calcium‐binding protein A9; sPLA2, secretory phospholipase A2; TNF‐α, tumor necrosis factor alpha, TNF‐B, tumor necrosis factor beta; YKL‐40, chitinase‐3‐like protein‐1.

Fourteen of the 37 reviewed studies measured inflammatory biomarkers in the chronic nic+ category (Figure 5). Interleukin (IL) 6 and 8 were measured the most out of all biomarkers reported in this category. Out of the five study results on IL‐6, two (40%) reported no change and three (60%) increase in inflammation. Out of the five times IL‐8 was reported, it did not change four (80%) and was increased one (20%) times. In these reviewed studies, use of EC increased 28 (26%) of 106 biomarkers and decreased 14 (13%); there was no change in 64 (60%). Of the three reported CV biomarkers, one (33%) increased, one (33%) decreased, and one (33%) had no change. Of 16 studies measuring pulmonary biomarkers, nine (56%) increased, and seven (44%) had no change. Of the 70 results reported on general inflammatory biomarkers, 14 (20%) increased, 11 (16%) decreased, and 45 (64%) had no change. Of the 17 oxidative stress biomarker reports, four (24%) increased, two (12%) decreased, and 11 (65%) had no change. A complete list of reviewed studies and measured biomarkers for this EC use category is presented in Table D3.

### 3.4 Chronic studies with unspecified nicotine‐containing EC devices

**Figure 6:**
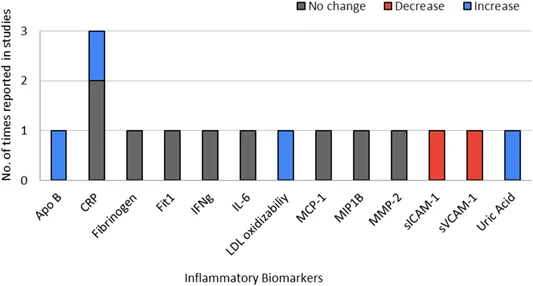
Inflammatory biomarker changes for reviewed chronic studies with unspecified use of nicotine‐containing e‐cigarette (EC) devices. Participants using ECs for more than 1 month were compared with baseline measurements or control groups. Each reported statistically significant increase or decrease was counted separately for a biomarker, and no statistically significant differences were counted as no change. Apo. B, apoprotein B; CRP, C‐reactive protein; FIT1, fat storage‐inducing transmembrane protein‐1; IFN‐γ, interferon gamma; IL‐6, interleukin 6; LDL, low‐density lipoprotein; MCP‐1, monocyte chemoattractant protein‐1; MIP‐1beta, macrophage inflammatory protein‐1 beta, MMP‐2, matrix metalloproteinase‐2, sICAM‐1, soluble intracellular adhesion molecule 1; sVCAM‐1, soluble vascular cell adhesion molecule‐1.

Four of the 37 reviewed studies measured inflammatory biomarkers in the nic+/− category because they did not provide sufficient information on participant exposure to nicotine or the concentration of nicotine in the ECs used by participants (Figure 6). C‐reactive protein (CRP) was the most commonly measured biomarker in this category, with results being reported three times. CRP was increased in only one of three studies and exhibited no change in two. In these reviewed studies, use of EC increased four (27%) of 15 biomarkers and decreased two (13%); there was no change in nine (60%). The two reported CV biomarkers were decreased. Only one study measured pulmonary biomarkers, and it reported no change. Of the 10 general inflammatory biomarkers reported in this category, two (20%) increased, and eight (80%) had no change. The two reported oxidative stress biomarkers were both increased. A complete list of reviewed studies and measured biomarkers for this EC use category is presented in Table D4.

## 4 DISCUSSION

In the current scoping review, we examined changes in general, cardiopulmonary, and oxidative stress‐related inflammatory biomarkers in healthy adults who use ECs. Thirty‐seven studies met our inclusion criteria. When EC devices containing nicotine were used <1 month, there were moderate increases CV and oxidative stress biomarkers. In general, 50% of CV biomarkers and 64% of oxidative stress markers were increased. When EC devices were used for more than a month, there were mixed results. Overall, regardless of EC use category, general inflammatory biomarkers had no change when compared with control groups. Because nicotine has been reported to increase the risk of multiple CV, respiratory, and gastrointestinal diseases,^18^ it is important to determine whether increasing levels of nicotine concentrate in ECs cause elevated inflammatory biomarkers and predispose users to increased risk of disease development. As such, it is also important to determine the effects of nicotine exposure after short‐term and long‐term EC use.

### 4.1 Acute EC use

In the current scoping review, inflammatory responses elicited by ECs varied depending on the duration of use and, if reported, the concentration of nicotine. When used for less than 1 month, nicotine‐containing ECs resulted in a majority of inflammatory biomarkers showing no change when compared with control groups. For CV biomarkers, there was mixed evidence supporting an increase or no change in these inflammatory mediators. In reviewed studies, CV biomarkers increased 11 times, decreased twice, and had no change nine times. Pulmonary biomarkers also had mixed results. Oxidative stress biomarkers were increased seven times and had no changes four times. Finally, general inflammatory biomarkers were measured to be increased in two of 13 studies and exhibit no change in 11. Taken together, these results suggested that nicotine‐containing ECs may predispose users to CV disease and do not upregulate general inflammatory processes in the short term.

When considering changes in specific biomarkers for reviewed studies, several notable findings were observed for this EC use category. FeNO was the most commonly measured overall, but this pulmonary biomarker had no change half the time it was measured. The next most common result for FeNO was a decrease. This finding was supported by previous research that reported decreased FeNO after the use of tobacco cigarettes.^19^ The second and third most commonly measured biomarkers were platelet selectin (P‐selectin) and CD40 ligand (CD40L), and both were increased in a majority of studies. In addition to FeNO, the only other biomarkers that had reported decreases in this category were Clara cell protein (CC16) and serum nitric oxide (NO). Although the decrease in CC16 was only reported once, it may corroborate the negative effects of nic+ EC use cigarettes on pulmonary inflammation. However, this finding should be interpreted cautiously because this biomarker was only measured in two studies. Serum NO was decreased in both studies that measured it. Similarly, tobacco cigarettes have been shown as major inhibitors of NO synthase, causing further CV dysfunction in users.^20^ The serum NO changes of the current scoping review suggest that EC users may experience a similar mechanism of inflammatory injury from tobacco cigarettes.

Similar results were reported by studies in the acute nic‐ EC use category. For CV, pulmonary, and oxidative stress biomarkers, there were no clear increases, decreases, or no change in biomarkers. Like the previous EC use category, a majority of the general inflammatory biomarkers were not different between control and experimental groups. More than half of the 11 general inflammatory biomarkers showed no change. Again, FeNO was the most commonly measured pulmonary biomarker and overall for this EC use category. In contrast to the acute nic+ category, there was no change in the three studies that measured it. Three biomarkers had reported decreases: CC16, NO metabolites, and protein‐bound 3‐chlorotyrosine/tyrosine ratio. The CC16 results in this category were the same as the acute nic+: one reported increase and one decrease. Surprisingly, the NO metabolites, an oxidative stress biomarker, were decreased in both measured instances. All other markers of oxidative stress were either increased or had no change. Several biomarkers that were measured once were increased in the nic‐ category. Two biomarkers that were measured twice, intercellular adhesion molecule 1 and CRP, were also increased. Despite study results, more data are required to determine whether there is an increased risk in general, CV, or oxidative stress biomarkers for users of ECs that do not contain nicotine. In particular, investigators interested in the short‐term effects of EC use should consider investigating changes in FeNO and other biomarkers have been implicated in such diseases as chronic obstructive pulmonary disease, asthma, stroke, hypertension, and other CV diseases.^21^, ^22^, ^23^

### 4.2 Chronic EC use

The current scoping review also found inflammation secondary to chronic EC use varied depending on which biomarker category was assessed. When used for more than 1 month, CV biomarker results for nicotine‐containing ECs were inconclusive; studies equally reported increases, decreases, or no change. Pulmonary biomarkers were increased in a majority of studies, but some reported no change. General inflammatory biomarkers overwhelmingly had no change between chronic nic+ EC users and controls. Oxidative stress biomarkers had mixed results, but most reported no change. Based on these results, several biomarkers stood out as potential candidates for future studies. Matrix metalloproteinase‐9 (MMP‐9) was a commonly measured biomarker in studies of long‐term EC use. This biomarker was reported four times: three increases and one no change. In the general inflammatory biomarker category, there were notable findings for several markers. IL‐6 and IL‐8 were the most commonly measured biomarkers. In almost a third of studies, IL‐6 was increased, but IL‐8 was increased in less than a quarter. Three other biomarkers were the second most commonly measured general inflammatory markers. One of these had no change in a majority of studies, and the other two had mixed results. Future studies of long‐term EC use should consider investigating such biomarkers as MMP‐9, IL‐6, and IL‐8. In particular, MMP‐9 and IL‐6 may be the best targets for prospective studies since they have been closely linked to the pathogenesis of chronic obstructive pulmonary disease, emphysema, rheumatoid arthritis, and various cancers.^24^, ^25^, ^26^, ^27^

For the nic+/− EC use category, there were some interesting results. All CV biomarkers were decreased in studies that did not specify exposure to nicotine. One pulmonary biomarker had no change when compared with control. General inflammatory biomarkers had no change in most studies. Oxidative stress biomarkers were only reported twice; both were increased. Because these results were inconclusive, we were unable to reach any conclusions about this EC use category. Clearly, more research with better reporting of methods is necessary.

## 5 LIMITATIONS

The current scoping review had several limitations that should be considered. Our inclusion criteria included studies with participants who had no confounding medical conditions or histories. Further, we had no exclusion criterion to remove studies that included healthy smokers as participants. Therefore, inflammatory biomarkers of healthy smoker participants may have influenced the reported results of reviewed studies. However, most reviewed studies performed rigorous history and physical exam screenings on participants before their use of ECs or before study outcome measurements were taken. A number of studies also required or encouraged participants to abstain from using products that could influence study outcomes, such as personal ECs, tobacco cigarettes, and caffeine. Another limitation of our review was the exclusion of studies that measured inflammatory biomarkers from participant samples not obtained from the sputum, respiratory gases, serum, or urine. For example, we excluded a few studies because they used participants' saliva for measurement purposes. After consideration, we decided that salivary measurements were not representative of overall CV, pulmonary, general inflammatory, and oxidative stress risk to EC users. Therefore, the current review does not include any salivary measurements in results or discussions.

## 6 CONCLUSION

Taken altogether, our scoping review suggested that short‐term use of nicotine‐containing ECs may result in increased CV inflammation, contributing to potential CV disease development in consumers. Short‐term use of ECs containing nicotine also caused an increase in the majority of oxidative‐stress biomarkers in participants, possibly predisposing consumers to the development of cancer, Alzheimer's disease, Parkinson's disease, and other neurologic conditions linked to high oxidative stress. Our scoping review also suggested that long‐term use of nicotine‐containing ECs resulted in no significant overall inflammatory changes of consumers, with the exception of MMP‐9 and IL‐6 being elevated in a majority of results. A more rigorous systematic review and meta‐analysis are necessary to corroborate our findings. Future studies could investigate the effect of long‐term EC use on MMP‐9 and IL‐6 levels to determine if consumers are at increased risk for related diseases such as asthma, rheumatoid arthritis, or cancer. Establishing a stronger link between EC use and inflammation‐mediated disease states would be greatly beneficial to physicians, healthcare professionals, and the general public.

## AUTHOR CONTRIBUTIONS

**Shawn Boss**: Conceptualization; data curation; formal analysis; investigation; methodology; project administration; resources; supervision; validation; visualization; writing—original draft; writing—review and editing. **Michael Bertolio**: Data curation; formal analysis; investigation; validation; writing—original draft; writing—review and editing. **Laura Lipke**: Data curation; methodology; supervision; validation; writing—original draft; writing—review and editing.

## CONFLICT OF INTEREST STATEMENT

The authors declare no conflict of interest.

## Data Availability

The data that support the findings of this study are available from the corresponding author upon reasonable request.

## APPENDIX A

**Search strategy for PubMed**.

((“Inflammation”[Majr] OR “inflamm*” [tw]) OR (“Acute‐Phase Reaction”[Mesh] OR “acute‐phase reaction” [tw]) OR (“Thromboinflammation”[Mesh] OR “thromboinflamm*” [tw]))) OR ((“Biomarkers”[Majr] OR “biomarker*” [tw]) OR (“endpoint*” [tw]) OR (“Antibodies, Antineutrophil Cytoplasmic”[Mesh]) OR (“Antigens, Differentiation, B‐Lymphocyte”[Mesh]) OR (“Antigens, Differentiation, Myelomonocytic”[Mesh]) OR (“Antigens, Differentiation, T‐Lymphocyte”[Mesh]) OR (“Biomarkers, Pharmacological”[Mesh]) OR (“Fibrinopeptide A”[Mesh]) OR (“Oligoclonal Bands”[Mesh])))) OR ((“Blood Proteins”[Majr] OR “blood protein*” [tw] OR “plasma protein*” [tw] OR “serum protein*” [tw] OR “Acute‐Phase Proteins”[Mesh] OR “acute‐phase protein*” [tw] OR “Immunoproteins”[Mesh] OR “immunoprotein*” [tw]))) OR ((“Glycoproteins”[Majr]))) OR ((“Stress, Physiological”[Majr]) OR (“Oxidative Stress”[Mesh] OR “oxidative stress*” [tw]))) OR (((“Free Radicals”[Majr] OR “free radicals” [tw]) OR (“Reactive Oxygen Species”[Mesh] OR “reactive oxygen specie*” [tw]) OR (“oxide” [tw])))) AND ((Electronic Nicotine Delivery Systems [MeSH] OR “electronic nicotine delivery system*” [tw] OR “electronic cigarette*” [tw] OR “e cigarette*” [tw] OR “e cigarette*” [tw] OR “e‐cig*” [tw])) limits humans and adults (average age 19–50)

## APPENDIX B

**Draft search strategy for medline using the Ovid platform**.

1. Inflammation/or acute‐phase reaction/or exp systemic inflammatory response syndrome/or exp thromboinflammation/
2. biomarkers/or antibodies, antineutrophil cytoplasmic/or exp antigens, cd/or antigens, differentiation, b‐lymphocyte/or antigens, differentiation, t‐lymphocyte/or antigens, differentiation, myelomonocytic/or biomarkers, pharmacological/or environmental biomarkers/
3. blood proteins/or exp acute‐phase proteins/or exp immunoproteins/
4. exp Glycoproteins/
5. stress, physiological/or exp oxidative stress/
6. free radicals/or exp reactive oxygen species/
7. exp Electronic Nicotine Delivery Systems/ae, mi, mo [Adverse Effects, Microbiology, Mortality]
8. (e‐cigarette* or e cigarette* or e‐cig* or e cig).mp. [mp=title, abstract, original title, name of substance word, subject heading word, floating subheading word, keyword heading word, organism supplementary concept word, protocol supplementary concept word, rare disease supplementary concept word, unique identifier, synonyms]
9. (1 OR 2 OR 3 OR 4 OR 5 OR 6) AND (7 OR 8)
10. limit 9 to (humans and “adult (19 to 50 years)”)

## APPENDIX C

**Data extraction form and inclusion/exclusion criteria for literature assessed**.

See Table C2

**Table C1:** Data extraction form used in the current scoping review.

Title
Title of paper/abstract/report that data are extracted from
Lead author, year published
Country in which study was published
Citation reference
Aim of study
Acute or chronic effects
Study design
Age range, gender(s)
Total number of participants
Electronic cigarette brand, nicotine +/‐ experimental groups (include concentration)
Category of biomarker(s) measured
Biomarker(s) measured
Results
Major conclusions
Study funding (if relevant)

**Table C2:** Inclusion and exclusion criteria for literature assessed in the current scoping review.

Inclusion criteria	Exclusion criteria
Biomarkers were measured through noninvasive collection of serum, urinalysis, imaging, and breath analyzer Study participants had no history of confounding illness Study participants were ages 18 and older Study was published in English In‐vivo measurements of biomarkers	Data from pediatric subjects (17 and younger) Studies including animal subjects Studies only using in‐vitro methods of measurement or analysis All salivary biomarkers

## APPENDIX D

**Documentation of biomarker results and respective literature**.

**Table D1:** Inflammatory biomarkers assessed for acute studies with nicotine‐containing e‐cigarette devices.

Investigators	Biomarker	Increased	Decreased	No change	Total No. of times reported
Benowitz (2020); Biondi‐Zoccai (2019); Carnevale (2016); Kotoulas (2020)	8‐iso‐prostaglandin	2		2	4
Chaumont (2019), (2020)	CC16	1	1		2
Antoniewicz (2016); Biondi‐Zoccai (2019); Mobarrez (2020); Nocella (2018)	CD40‐ligand (CD154)	3		1	4
Kerr (2019)	E‐selectin (CD62E)	1			1
Mobarrez (2020)	EC derived EVs (PS + CD62E + )	1			1
Kerr (2019); Staudt (2018)	EMP	1		1	2
Antoniewicz (2016)	Endothelial‐derived MV (CD144)			1	1
Mobarrez (2020)	Platelet (CD41) derived EV	1			1
Antoniewicz (2016); Brożek (2019); D'Ruiz (2016); Flouris (2013); Kotoulas (2020); Palamidas (2017); Schober (2014); Vardavas (2012)	FeNO	1	3	4	8
Antoniewicz (2016)	HMGB‐1			1	1
Chaumont (2018)	Homocitrulline/lysine ratio			1	1
Kerr (2019)	ICAM			1	1
Kotoulas (2020)	IL‐17A			1	1
Kotoulas (2020)	IL‐1β			1	1
Kotoulas (2020)	IL‐5			1	1
Benowitz (2020); Kotoulas (2020)	IL‐6	1		1	2
Benowitz (2020); Kotoulas (2020)	IL‐8	1		1	2
Antoniewicz (2016)	Leukocyte (CD45) derived EVs			1	1
Kotoulas (2020)	LTB4			1	1
Ikonomidis (2018)	MDA	1			1
Antoniewicz (2016)	Monocyte‐derived MV (CD14)			1	1
Chaumont (2018)	MPO	1			1
Biondi‐Zoccai (2019); Carnevale (2016)	Nox2‐derived peptide	2			2
Antoniewicz (2016)	Nuclear content (SYTO 13 dye)			1	1
Antoniewicz (2016); Biondi‐Zoccai (2019); Kerr (2019); Mobarrez (2020); Nocella (2019)	P‐selectin (CD62P)	3		2	5
Kerr (2019)	PECAM			1	1
Antoniewicz (2016)	Platelet‐derived MV (CD41)			1	1
Kerr (2019)	PMP	1			1
Chaumont (2018)	Protein‐Bound‐3‐Chloro‐ thyrosine/Tyrosine ratio			1	1
Biondi‐Zoccai (2019)	Serum H2O2	1			1
Biondi‐Zoccai (2019); Roberto Carnevale (2016)	Serum NO		2		2
Kotoulas (2020)	TNF‐α			1	1
Kerr (2019)	VCAM			1	1

Abbreviations: CC16, Clara cell protein 16; EC, electronic cigarettes; EMP; endothelial microparticles; EV, extracellular vesicles; FeNO, fraction of exhaled nitric oxide; HMGB‐1, high mobility group box 1 protein; ICAM, intracellular adhesion molecule; IL, interleukin; LTB4, leukotriene B4; MDA, malondialdehyde; MPO, myeloperoxidase; PECAM, platelet endothelial cell adhesion molecule; PMP, platelet microparticle; TNF, tumor necrosis factor; VCAM, vascular cell adhesion molecule.

**Table D2:** Inflammatory biomarkers assessed for acute studies without nicotine‐containing e‐cigarette devices.

Investigators	Biomarker	Increased	Decreased	No change	Total no. of times reported
Chatterjee (2019)	ASC	1			1
Chaumont (2019), (2020)	CC16	1	1		2
Mobarrez (2020)	CD40L	1			1
Chatterjee (2019), (2021)	CRP	2			2
Mobarrez (2020)	EC derived Evs (PS + CD62E + )			1	1
Kerr (2019)	EMP			1	1
Mobarrez (2020)	EV (PS + CD41 + + CD62P)			1	1
Ferrari (2015); Palamidas (2017); Schober (2014)	FeNO			3	3
Chatterjee (2021)	HMGB‐1	1			1
Chaumont (2018)	Homocitrulline/lysine ratio			1	1
Song (2020)	IFN‐γ			1	1
Song (2020)	IL‐12p70			1	1
Song (2020)	IL‐1β			1	1
Song (2020)	IL‐2			1	1
Song (2020)	IL‐6			1	1
Song (2020)	IL‐8			1	1
Ikonomidis (2018)	MDA	1			1
Chaumont (2018)	Myeloperoxidase			1	1
Chatterjee (2019), (2021)	Nitric oxide metabolites		2		2
Chaumont (2018)	Protein‐bound‐3‐chloro‐thyrosine/tyrosine ratio		1		1
Chatterjee (2019), (2021)	sICAM‐1	2			2
Song (2020)	TNF‐α			1	1

Abbreviations: ASC, apoptosis‐associated speck‐like protein; CC16, Clara cell protein 16; CD40L, CD40 ligand; CRP, C‐reactive protein; EC EVs, endothelial cell‐derived extracellular vesicles; EMP, endothelial microparticles; FeNO, fraction of exhaled nitric oxide; HMGB‐1, high mobility group box 1 protein; IFN‐γ, interferon gamma; IL, interleukin; MDA, malondialdehyde; sICAM‐1, soluble intercellular adhesion molecule 1; TNF‐α, tumor necrosis factor alpha.

**Table D3:** Inflammatory biomarkers assessed for chronic studies with nicotine‐containing e‐cigarette devices.

Investigators	Biomarker	Increased	Decreased	No change	Total no. of times reported
Gupta (2021)	13‐HODE		1		1
Wetherill (2022)	18F‐NOS BPND	1			1
Singh (2019)	4‐hydroxynonenal			1	1
Singh^a^ (2019)	8‐isoprostane	1		1	2
Singh (2019)	8‐oxo‐dG	1			1
Gupta (2021)	9‐HODE		1		1
Tsai (2019)	ASC	1			1
Reidel (2018)	Azurocidin 1	1			1
Reidel (2018)	Calprotectin	1			1
Tsai (2019)	Caspase‐1			1	1
Singh^a^ (2019)	CC10			2	2
Singh^a^ (2019)	CC16			2	2
Sayed (2021)	CD40L		1		1
Reidel (2018)	Coronin‐1	1			1
Kelesidis (2020)	COS in B cells			1	1
Kelesidis (2020)	COS in CD45			1	1
Kelesidis (2020)	COS in monocytes	1			1
Kelesidis (2020)	COS in neutrophils			1	1
Kelesidis (2020)	COS in NK cells			1	1
Kelesidis (2020)	COS in T cells			1	1
Hickman (2022); Singh (2019)	CRP			2	2
Singh (2019)	CXCL1		1		1
Singh (2019)	CXCL2			1	1
Singh^a^ (2019)	EN‐rage			2	2
Sayed (2021); Singh (2019)	Eotaxin			2	2
Meo (2019)	FeNO			1	1
Singh (2019)	Fibrinogen			1	1
Singh (2019)	G‐CSF		1		1
Singh^a^ (2019)	Galectin‐3			2	2
Sayed (2021); Singh^a^ (2019)	GM‐CSF		1	2	3
Singh (2019)	ICAM‐1	1			1
Sayed (2021)	IFNa2		1		1
Sayed (2021); Singh^a^ (2019); Song (2020)	IFNY	1	2	1	4
Sayed (2021)	IL‐15		1		1
Singh (2019)	IL‐1α			1	1
Sayed (2021)	IL‐7		1		1
Song (2020)	IL12p70			1	1
Singh^a^ (2019); Song (2020); Tsai (2019)	IL1B	2		2	4
Sayed (2021); Song (2020)	IL2		1	1	2
Perez (2020); Sayed (2021); Singh^a^ (2019); Wetherill (2022)	IL6	3		2	5
Perez (2020); Sayed (2021); Singh^a^ (2019); Wetherill (2022)	IL8	1		4	5
Singh (2019)	Malondialdehyde			1	1
Sayed (2021); Singh (2019)	MCP‐1			2	2
Reidel (2018)	MSPβ			1	1
Singh^a^ (2019)	MIP‐1α			2	2
Singh^a^ (2019)	MIP‐1β			2	2
Ghosh (2019)	MMP‐2	1			1
Ghosh (2019); Reidel (2018); Singh^a^ (2019)	MMP‐9	3		1	4
Reidel (2018); Singh^a^ (2019)	MPO	1		2	3
Reidel (2018)	MUC5AC	1			1
Reidel (2018)	MUC5B			1	1
Ghosh (2019); Reidel (2018)	NEUT. elastase	2			2
Reidel (2018)	Nucleobindin‐1			1	1
Singh^a^ (2019)	PAI‐1/serpine‐1			2	2
Reidel (2018)	PAD4	1			1
Reidel (2018)	Proteinase 3	1			1
Singh^a^ (2019)	RAGE		1	1	2
Singh^a^ (2019)	RANTES			2	2
Singh^a^ (2019)	S100A8			2	2
Singh^a^ (2019)	S100A9			2	2
Gupta (2021)	sPLA2			1	1
Singh (2019)	Thromboxane B2			1	1
Perez (2020); Sayed (2021); Singh (2019); Song (2020)	TNFa		1	3	4
Sayed (2021)	TNFβ	1			1
Perez (2020)	YKL‐40	1			1

Abbreviations: 18F‐NOS BPND, 18F‐6‐(1/2)(2‐fluoro‐propyl)‐4‐methylpyridin‐2‐amine nondisplaceable binding potential; ASC, apoptosis‐associated speck‐like protein; CC, Clara cell protein; COS, cytoplasmic oxidative stress; CRP, C‐reactive protein; CSF, colony stimulating factor; CXCL1, chemokine (C‐X‐C motif) ligand 1; FeNO, fraction of exhaled nitric oxide; HODE, hydroxyoctadecadienoic acid; ICAM, intracellular adhesion molecule; IFN, interferon; IL, interleukin; MCP‐1, monocyte chemoattractant protein‐1; MIP, macrophage inflammatory protein; MMP, matrix metalloproteinase; MPO, myeloperoxidase; MSPB, microseminoprotein beta; MUC5AC, mucin 5AC; MUC5B, mucin 5B; NEUT elastase, neutrophil elastase; PAI‐1, plasminogen activator inhibitor 1; PAD4, peptidyl arginine deiminase 4; RAGE, receptor for advanced glycation endproducts; RANTES, regulated upon activation, normal T cell expressed and presumably secreted; S100A8, S100 calcium‐binding protein A8; S100A9, S100 calcium‐binding protein A9; TNF, tumor necrosis factor; YKL‐40, chitinase‐3‐like protein‐1.

^a^ Multiple methods of measurement (serum, urine, etc.) used in study of biomarker.

**Table D4:** Inflammatory biomarkers assessed for chronic studies with unspecified nicotine‐containing e‐cigarette devices.

Investigators	Biomarker	Increased	Decreased	No change	Total no. of times reported
Hickman (2022); Moheimani (2017); Moon (2020)	CRP	1		2	3
Hickman (2022)	IL‐6			1	1
Hickman (2022)	MMP‐2			1	1
Hickman (2022)	sICAM‐1		1		1
Hickman (2022)	sVCAM‐1		1		1
Hickman (2022)	IFN‐γ			1	1
Hickman (2022)	MCP‐1			1	1
Hickman (2022)	MIP1B			1	1
Hickman (2022)	Fit1			1	1
Moheimani (2017)	LDL oxidizability	1			1
Moheimani (2017)	Apo B	1			1
Moheimani (2017)	Fibrinogen			1	1
Moon (2020)	Uric Acid	1			1

Abbreviations: CRP, C‐reactive protein; FIT1, fat storage‐inducing transmembrane protein‐1; IFN, interferon; IL, interleukin; LDL, low‐density lipoprotein; MCP‐1, monocyte chemoattractant protein‐1; MIP, macrophage inflammatory protein; MMP, matrix metalloproteinase; sICAM‐1, soluble intracellular adhesion molecule 1; sVCAM‐1, soluble vascular cell adhesion molecule 1.
